# Network-Based Biomarkers in Alzheimer’s Disease: Review and Future Directions

**DOI:** 10.3389/fnagi.2014.00012

**Published:** 2014-02-04

**Authors:** Jaime Gomez-Ramirez, Jinglong Wu

**Affiliations:** ^1^Autonomous Systems Laboratory, Universidad Politécnica de Madrid, Madrid, Spain; ^2^Biomedical Engineering Laboratory, Okayama University, Okayama, Japan

**Keywords:** Alzheimer’s disease, network degeneration hypothesis, network-based biomarkers, default-mode network DMN, resting-state functional connectivity

## Abstract

By 2050 it is estimated that the number of worldwide Alzheimer’s disease (AD) patients will quadruple from the current number of 36 million people. To date, no single test, prior to postmortem examination, can confirm that a person suffers from AD. Therefore, there is a strong need for accurate and sensitive tools for the early diagnoses of AD. The complex etiology and multiple pathogenesis of AD call for a system-level understanding of the currently available biomarkers and the study of new biomarkers via network-based modeling of heterogeneous data types. In this review, we summarize recent research on the study of AD as a connectivity syndrome. We argue that a network-based approach in biomarker discovery will provide key insights to fully understand the network degeneration hypothesis (disease starts in specific network areas and progressively spreads to connected areas of the initial loci-networks) with a potential impact for early diagnosis and disease-modifying treatments. We introduce a new framework for the quantitative study of biomarkers that can help shorten the transition between academic research and clinical diagnosis in AD.

## Introduction

A biomarker is a parameter that can be used as an indicator of normal biological processes, pathogenic processes, or pharmacological responses to therapeutic drugs (Biomarkers Definitions Working Group, 2001). In Alzheimer’s disease (AD), potential biomarker information comes from multiple sources, including clinical tests for memory impairment, bodily fluid or tissues, neuroimaging, and smell tests among others. AD biomarkers are typically assumed to belong to the following two categories: *biofluid analytes*, e.g., cerebrospinal fluid (CSF), peripheral blood samples such as urine and *imaging measures*, e.g., magnetic resonance imaging (MRI), magnetic resonance spectroscopy (MRS), or positron emission tomography (PET) (Henriksen et al., 2014). At present there are five well-established AD biomarkers: two are CSF analytes that measure abnormal protein aggregates – low level of CSF amyloid-beta and elevated level of both total and phosphorylated CSF tau protein; and three imaging biomarkers – the Pittsburgh compound-B PIB PET tracer for amyloid-beta deposition, for which MRI scans may detect atrophied sensible brain areas; and Fludeoxyglucose FDG PET to quantify abnormal neuronal glucose consumption (Jack, 2012).

The diagnostic criteria for AD has not been modified since its original formulation in 1984 until it was recently updated in 2010 (Dubois et al., 2010). In the original criteria, AD was strictly diagnosed on a clinical basis (McKhann et al., 1984). Other sources of information such as imaging lacked a positive diagnostic role. New diagnostic criteria reckons AD as a complex disorder characterized by a gradual and progressive pathogenesis, with three phases – preclinical or asymptomatic, prodromal or mild cognitive impairment (MCI), and overt dementia (Dubois et al., 2007; Albert et al., 2011; Sperling, 2011). Despite technological and conceptual advances in AD, we are still lacking preventive therapies to delay the onset of AD as well as disease-modifying treatments. Despite the strong need for early diagnose of AD, and the fact that biomarkers have proved useful in correlating with the different stages in which the disease unfolds, CSF and imaging biomarkers still play a surprisingly minor role in clinical diagnosis. They are, however, increasingly prominent in clinical trials and academic research.

There is a growing consensus between clinical researchers that the application of biomarkers should follow a multi-modal and integrative approach. Truly predictive models of disease progression need to take into account the combined effects of biomarkers interactions at the individual subject level. Unfortunately however, few studies have specifically addressed the issue of the integration of different biomarkers for efficient and quantitative diagnostics. Furthermore, it has been particularly difficult to link findings on molecular biomarkers to early stages of the neurodegenerative disease, and no real groundbreaking discovery in imaging-based biomarkers has been produced. Thus, there is a lack of novel therapeutic approaches that efficiently target the underlying mechanisms and disease progression of AD (Corbett and Ballard, 2012). There is clear evidence that AD and other neurodegenerative disorders evolve at the systems level (Eidelberg and Martin, 2013) and that biomarkers – molecular, imaging, or CSF – need to be considered with a holistic point of view. Functional imaging may help us understand disease-related changes in interconnected brain areas. In this regard, functional imaging techniques unburdened of subject compliance such as RS-functional magnetic resonance imaging (fMRI) and TMS/EEG, are being extensively used for biomarkers discovery in neurodegenerative disorders.

In this review, we provide a brief panoramic view on recent research on the discovery of AD biomarkers, putting special emphasis on neuroimaging biomarkers derived from functional connectivity data in resting state, that is, the subject is not performing an explicit task. Network-based biomarkers are introduced, and we provide a new framework for the quantitative study of biomarkers that can help shorten the transition between academic research and clinical diagnosis in AD.

## AD Biomarkers

Clinical tests for AD diagnosis involve subjective reasoning by experienced practitioners. Episodic memory impairment has little or no relevance in early diagnosis, but it still remains the core diagnostic criterion. Current diagnostic criteria (DSM-IV and NINCDS-ADRDA) have high sensitivity but low specificity (Knopman et al., 2001). The delay from symptoms to diagnosis is 20 months on average in the EU, and 36 months in the UK (Mattila et al., 2012). Furthermore, molecular pathomechanisms of AD become active for several years before symptoms such as cognitive impairment manifests itself.

Blood samples are a non-invasive and cost-effective technique for the identification of plasma biomarkers that has proven useful in distinguishing individuals with AD from cognitively healthy control subjects (Doecke et al., 2012). Plasma biomarkers can be used to extract metabolomics (Trushina et al., 2013) and proteomics biomarker signatures in AD (Hye et al., 2006). Contrary to diagnostic tools like CSF and PET, plasma amyloid-beta measurements are neither invasive nor expensive. Plasma Aβ40 and Aβ42 can be measured in peripheral blood, but they cannot be used in AD identification. Vanderstichele et al. (2000) found no differences in Aβ42 levels between controls and patients with AD. Further work is required before plasma amyloid-beta measurements are unanimously regarded as clinically useful (Mayeux and Schupf, 2011; Toledo et al., 2013).

Using Smell tests to detect hyposmia is another example of inexpensive biomarker in AD (Kjelvik et al., 2007). However, the reduced capability to detect odors shown in AD may be more an effect of the cognitive decline characteristic of the disease than a symptom with predictive value (Serby et al., 1991).

Neuroimaging biomarkers in AD measure brain signals at both mesoscopic (MRI) and macroscopic scales (fMRI, MRS, and PET). Morphometric analysis with MRI data (e.g., atrophy in medial temporal lobes, specifically in the hippocampus and entorhinal cortex) is a well-known marker of disease progression in AD. Hippocampus atrophy correlates with neuronal loss and therefore MRI biomarkers could be used in proof-of-the-concept studies to distinguish between disease-modifying and symptomatic treatment effects (Saumier et al., 2009; Hampel et al., 2011). PET neuroimaging allows us to collect molecular information. PET image analysis can provide evidence of the accumulation of amyloid-beta plaques that is independent from structural brain changes. It also provides evidence of a reduction of glucose metabolism in the parietal and temporal lobe regions that are involved in memory and executive function (Habeck et al., 2012). Both structural MRI and FDG-PET imaging reflect the effects of the disease progress in symptomatic stages, however it is the diagnosis in AD’s asymptomatic stages that remains to be solved. Molecular pathomechanisms, such as the accumulation of amyloid plaque, become active several years before cognitive deficit manifest. Furthermore, amyloid-beta is not specific to AD, but may also be found in normal aging.

## Resting-State fMRI

Functional magnetic resonance imaging allows us to assess functional connectivity mapping at high temporal resolution by means of correlations in the blood-oxygen-level-dependent (BOLD) signal in spatially distant brain regions. Since the seminal work of Biswal (Biswal et al., 1995), task-free or resting-state fMRI (R-fMRI) has been successfully incorporated into the functional MRI imaging repertoire, and represents a comprehensive alternative to the task-based approach. R-fMRI experiments are considerably less demanding for the subject, which makes this technique especially attractive to brain dementia researchers, as it is relatively free of subject compliance and training demands. R-fMRI measures the spontaneous or intrinsic brain activity in terms of low-frequency (<0.1 Hz) BOLD fluctuations. Fluctuations in the BOLD signal measured in humans in resting state represent the neuronal activity baseline and shape spatially consistent patterns (Fransson, 2005; Raichle and Gusnard, 2005). The systematic study of those patterns using correlation analysis techniques has identified a number of resting-state networks, which are functionally relevant networks found in subjects in the absence of either goal directed-task or external stimuli. Despite the variability in the data acquisition protocols, statistical data analysis, and groups of subjects employed, resting-state networks have been consistently reported in multiple studies. There are at least eight commonly identified resting-state networks: the primary sensorimotor network, the primary visual and extra-striate visual network, bilateral temporal/insular, and anterior cingulate cortex regions, left and right lateralized networks consisting of superior parietal and superior frontal regions, and the default-mode network (DMN) (Van den Heuvel and Hulshoff Pol, 2010).

The DMN is a specific anatomically defined brain system that is preferentially active when individuals are focused on introspective activities such as autobiographical memory retrieval, rather than on the external environment (Buckner et al., 2008). A number of studies indicate that the default network is also relevant for understanding mental disorders including depression (Sheline et al., 2009), autism (Washington et al., 2013), and AD. Studies show a decrease in DMN functional connectivity in normal aging, MCI and AD (Hafkemeijer et al., 2012). Functional connectivity of the DMN may prove to be a sensitive and specific biomarker for mild AD (Greicius et al., 2004; Balthazar et al., 2014).

The visual identification of the overall connectivity patters in R-fMRI has been assessed using either model-based or model-free approaches. In the former, statistical parametric maps of brain activation are built upon voxel-wise analysis location (Wang et al., 2009; Faria et al., 2012). This approach has been successful in the identification of motor networks, but it shows important limitations when the seed voxel cannot be easily identified, for example in brain areas with unclear boundaries such as cognitive networks involved in language or memory. Independent component analysis (ICA) (Comon, 1994; Stone, 2002), on the other hand, is a model-free approach that allows separating resting fluctuations from other signal variations, resulting in a collection of spatial maps, one for each independent component, that represent functionally relevant networks in the brain. While ICA has an advantage over model-free methods that it is unbiased, that is, it does not need to posit a specific temporal model of correlation between regions of interest (ROI), the functional relevance of the different components is still computed relative to their resemblance to a number of networks based on criteria that are not easily formalized (Friston, 1998). More recently researchers using graph-theory based methods have been able to not only visualize brain networks, but also to quantify their topological properties as well (He et al., 2009; Wang et al., 2010). Graph-theory provides a formal and rigorous framework to quantitatively analyze the connectivity pattern, at either a local or global level, underlying cognitive networks. How these network properties are modified during normal development, aging, or pathological conditions is addressed in the next section.

## R-fMRI and AD

Altered resting-state functional connectivity patterns have been shown in an impressive range of pathologies and conditions – AD, schizophrenia, multiple sclerosis, Parkinson’s disease, depression, autism, and attention deficit/hyperactivity disorder – see (Lee et al., 2013) for a review on clinical applications. In the context of AD, both amyloid-beta and tau pathologies affect DMN integrity before the clinical onset of the disease (Li et al., 2013; Wang et al., 2013). DMN regions such as the precuneus and the posterior cingulate are selectively vulnerable to amyloid-beta deposition (Sperling et al., 2010). AD weakens structural and functional connectivity between the cingulate cortex and other regions within the DMN, which is consistent with the reduction in metabolic activity and atrophy observed with FDP-PET and volumetric MRI, respectively within the DMN (Zhu et al., 2013). Patients with severe AD show decreased connectivity between distant brain regions (Liu et al., 2013). Interest in understanding the pathomechanisms of tau-mediated neurodegeneration has been fostered by the failure of amyloid-beta therapies to prevent neurodegeneration by Aβ removal. Tau abnormalities have been found to be more closely related to cognitive dysfunction than Aβ (Yoshiyama et al., 2012). Tau deposition is initially located in the medio-temporal lobe to spread later to lateral temporal and frontal parietal areas. This orderly progression found in hypophosphorylated tau maps the regional specificity in the deployment of symptoms in AD, i.e., episodic memory loss in the MTL is followed by semantic memory loss in lateral temporal cortex to aphasic symptoms in parietal cortex (Pievani et al., 2011).

Functional imaging has been successfully used in population selection in cross-sectional studies to classify between normally aging, MCI, and AD subjects (Rombouts et al., 2005; Damoiseaux, 2012). R-fMRI can be also used to track AD progression in longitudinal studies. For example, in Damoiseaux et al. (2012) it is shown that functional connectivity in default-mode subnetworks decreases in AD patients compared to healthy controls. Resting-state functional connectivity can help detect early manifestations of genetic effects related to AD. For instance, in (Sheline et al., 2010) cognitive normal individuals were categorized into PIB− (no evidence of brain amyloid) and PIB+ (PET evidence of amyloid deposition) and compared with AD patients using resting-state functional connectivity. The study showed that the PIB+ and AD groups share similar modifications in both functional and effective connectivity. Thus, R-fMRI can be used to detect early manifestations of genetic effect, e.g., amyloid deposition in APOE4 carriers, and therefore holds great potential in early diagnosis and disease-modifying strategies. It goes without saying that like any technique, R-fMRI has advantages and disadvantages. fMRI measures the BOLD signal, which is an indirect measure of neural activity and it is susceptible to several imaging artifacts and has, in general, worse temporal resolution than EEG and MEG, and spatial resolution that is not as good as more invasive procedures such as single-unit electrodes. The analysis and interpretation of R-fMRI data is particularly challenging, and further work is still required to address complex issues like network identification, effective connectivity between brain networks, detecting AD risk groups, etc. For a review on the progress and pending problems of statistical approaches to analyzing R-fMRI, see Cole et al. (2010).

## Network-Based Biomarkers

Contrary to other conditions such as brain injury whose onset can be tracked both in location and time, late sporadic AD – the most common form of dementia and two orders of magnitude more frequent than inherited AD (Bateman et al., 2012) – has a gradual onset that lacks a specific location or temporal window. Experimental studies based on neuropathology, neuroimaging, and transgenic animal models suggest that neurodegeneration relates to neural network dysfunction. Disease-vulnerable intrinsic functional networks are not diffuse or random (Sanz-Arigita et al., 2010), however, researchers are still uncertain about the specific way in which neurodegeneration spreads beyond the sites of initial impairment. The network degeneration hypothesis (Seeley et al., 2009) – disease starts in small network assemblies, to progressively spread to connected areas of the initial locus – supports the view that neurodegenerative disorders can be study as connectivity disorders. In this light, AD can be understood as a disconnection syndrome in which the structural and functional connectivity of large-scale networks is progressively modified by molecular pathomechanisms that are not fully understood.

A diagnostic biomarker, in order to be considered as such, should reflect a core pathogenic process. The established biomarkers in AD hold this promise as they measure, for example, amyloid-beta and tau deposition levels, which are responsible for the formation of senile plaques and neurofibrillary tangles. However, it is far from clear whether amyloid and tau deposition are etiologically linked to memory deficits or they rather reflect secondary effects of a different pathogenic mechanism (Eidelberg and Martin, 2013). AD is a complex and multifactorial condition and so “secondary processes” such as oxidative stress, immune responses, or inflammation and how they interact with core pathogenic mechanisms need to be properly understood.

The discovery of AD biomarkers must go beyond detecting abnormal protein deposition levels and be able to monitor both disease progression and treatment effects in a coherent and integrative way. To that end, a network-based approach for biomarker discovery is required. Erler and Linding (2010) argue that biomarkers should be deployed as network models themselves. The rationale behind this idea is that biomarker discovery needs to take into account the network state and the biological context in which the network evolves, rather than focus on individual nodes or events, e.g., phosphorylation. A network-based approach for biomarker discovery is also being fostered in complex diseases such as cancer and diabetes (Ahn et al., 2006).

The multifactorial pathogenesis of complex diseases such as AD is at odds with the current implementation of biomarkers which are single-dimensional. Thus, we propose to redefine biomarker as *a network model that can be used as an indicator of normal (including adaptive) biological processes, pathogenic processes, or pharmacological responses to therapeutic drugs*. Under this definition, biomarkers are multidimensional, as they are embedded into a network model in which network parameters, that represent normal or pathological processes but also adaptive responses, can be characterized. This new definition of biomarker allows us to quantify adaptive processes triggered by early pathogenic events, fostering an integrative and multidimensional approach of use in AD early diagnose. For example, it is unclear if, as the disease progresses, functional connectivity in large neural systems is attenuated, e.g., in the DMN (Wu et al., 2011; Liu et al., 2013; Zhu et al., 2013) or on the contrary, AD may induce an increase in functional connectivity that compensates for the disease related atrophy of affected regions (Sanz-Arigita et al., 2010). An increase in focal frontal connectivity and heightened hippocampal activation during early stages of AD has been reported in Dickerson et al. (2004). Functional disruption has been observed in the prodromal stage or even earlier and therefore a characterization of this imaging phenotype has potential impact in early prevention and disease-modifying therapies. The relationship between brain development, aging and disease and brain connectivity is not univocal, but instead involves a number of complex mechanisms that alter the network topology in multiple ways. The mechanisms that mediate in the increase in functional connectivity observed in prodromal AD are in dispute. There are several potential explanations for this phenomenon. For example, the increase in connectivity in the early phases of AD could reflect compensatory effects to neutralize the disruption in functional integrity, or represent some form of glutamate receptor-mediated excitotoxicity (Wu et al., 1995). An interesting hypothesis borrowed from economic theory is that early network alterations can be interpreted as a discount factor that anticipates the expectation of pending functional network integrity deterioration.

Combining existing biomarkers poses important challenges not only in terms of intelligibility due to the heterogeneous and complex nature of biomarker data, but also in terms of cost of data extraction, e.g., expensive SPECT or MRI can not be used in subjects with metal implants, and genetic mutations account for only a small percentage of AD cases (Bertram and Tanzi, 2004). Truly predictive models of disease progression need to take into account the combined effects of biomarkers interactions at the individual subject level. Few studies however, have specifically addressed the issue of the integration of different biomarkers (Gomar et al., 2011). The long sought goal of early diagnosis of AD necessarily passes by the integration of existing biomarkers and the discovery of new ones. Network-based biomarkers provide a unifying approach for AD biomarker discovery and testing. Graph-based network analysis allows to quantitatively characterize the global organization of the brain and to integrate heterogeneous data in a “neutral” and general mathematical body.

## A Network-Based Approach in AD Biomarkers

Biomarkers can be compounds obtained from bodily fluids or tissues, or technically derived correlates of pathophysiological events. While three of the five most important AD biomarkers are imaging-based, functional neuroimaging is absent in current diagnostic criteria.

Markers of alterations in resting-state functional connectivity networks can discriminate between AD patients and healthy elderly people with a satisfactory level of sensitivity and specificity. Functional connectivity analysis of the DMN has great potential as network biomarker able to objectively quantify asymptomatic and prodromal stages of the disease and as secondary endpoint in multicenter clinical trials in AD (Chhatwal et al., 2013). The study of AD biomarkers with R-fMRI imaging, however, has focused on detecting alterations in specific networks such as the DMN and finding abnormal levels of protein deposition, metabolic disruption, and atrophy within the DMN. A system-level understanding of the dependencies that exist among the different biomarkers has not been achieved. The advent of “Big Data” science makes it possible to share large amount of data with unprecedented processing capability. The Alzheimer’s disease neuroimaging initiative (ADNI) makes access to clinical imaging and biomarker data freely available to researchers worldwide. The whole genome sequences of the 800 individuals enrolled in the ADNI will be soon available through the Global Alzheimer’s Association Interactive Network (GAAIN).

The much-needed insight into the pathomechanisms that mediate in AD will benefit from the construction of probabilistic networks from large databases of AD biomarkers that systematically capture the probabilistic dependencies among biomarkers. Once the network or networks are built, a supervised classification algorithm can be used to classify new subjects within different classes, for example healthy and AD. Thus, in a training set of patients diagnosed as healthy or AD, we first build the generative graphs – *M*_H_ and *M*_AD_ – containing biomarker dependencies of healthy and AD subjects, respectively, to later perform a classification inference, that is, estimate the likelihood that *M*_H_ or *M*_AD_ has generated new data, i.e., a new subject to be diagnosed.

**Figure 1:**
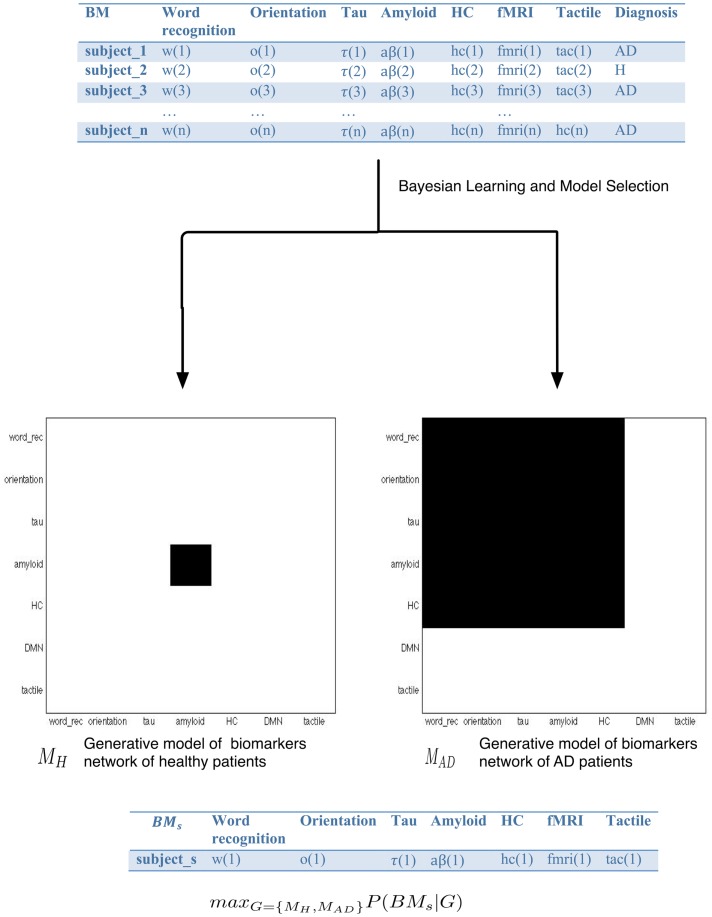
**Seven biomarkers of interest are listed in BM**. For convenience, we assume that BM is a binary vector, that is, BM(*i*) = 0,1. For example, if the measurement of the biomarker Word recognition reaches the positive threshold BM(1) = 1, if not, BM(1) = 0. The table in the top of the figure shows the training set S consisting of *n* samples or subjects with their biomarkers BM, and diagnosed as AD or healthy. The data in the table can be summarized via the construction of generative networks, one for each diagnostic category, in our example H and AD. There is a number of possible network structures that can characterize the training set, so the generative networks *M*_H_ and *M*_AD_ are the result of model selection. The diagnosis of new patients can be thus be addressed via the computation of the probability that the new data, BM_s_ is generated by the biomarker network that captures the dependencies among biomarkers in healthy subjects or by the biomarker network of healthy subjects.

Let us see this with an example. Figure 1 shows a classification procedure for AD using a biomarker network-based approach. BM is a list of AD biomarkers considered in this example, BM = (w, o, τ, aβ, hc, fc, tac). For convenience, we assume that BM takes discrete values, that is, BM*_i_* = 1 when biomarker *i* reaches the threshold of positivity. Thus, w (Word recognition) and o (Orientation) are neuropsychological markers included in the ADAS-Cog (Alzheimer Disease Assessment Scale-Cognitive) (Rosen et al., 1984), τ and Aβ are CSF biomarkers that indicate whether the protein deposition is relevant, hc (hippocampus) is equal to 1 when a significant reduction of the hippocampus volume is found, fc (functional connectivity) indicates whether regions in, for example, the DMN such as the precuneus or the posterior cingulate cortex, has functional connectivity alterations reported in the literature or any other pattern that we want to be tested against other biomarkers. The tactile biomarker (tac) is an inexpensive marker of cognitive and motor decline of interest in AD found in our laboratory (Yang et al., 2010). This list of biomarkers can be extended with others, e.g., smell, epigenetic, blood, genetic, etc., with the caveat that a large number of parameters need even larger data sets in order to avoid having an overwhelming choice of networks that are potentially good at explaining the data.

The training data set *S* is ideally composed of a large number of diagnosed subjects with the BM vector of biomarker information for each one. Thus, the training set is given by *S* = [(BM_1_, *y*)(BM_2_, *y*),…(BM*_n_, y*)], where BM*_i_* is the vector containing the biomarkers measured in patient *i*, and *y* represents the diagnostic class in which a subject can be classified, e.g., Healthy or AD. Now, we want to build a probabilistic network that captures dependencies among the biomarkers for each diagnostic class. For example, if the training data set contains biomarker information of *n* subjects diagnosed as healthy or AD [*y* = (*y*_H_, *y*_AD_)], two generative biomarker networks – *M*_H_ and *M*_AD_ – need to be built. This approach is entirely different to conventional AD biomarker studies, summarized above, that treat biomarkers as quantities that reflect relevant biological processes whose correlations with other biomarkers need to be investigated through heuristics methods (Table 1). An interesting improvement in the quantification and integration of AD biomarkers aiming to improve the efficiency and of AD diagnosis can be found in Mattila et al. (2011). A supervised classifier is implemented via a disease state index (DSI) that compares the biomarker measurements of new patients with previously diagnosed patients’ biomarkers. Thus, the DSI is an aggregate measure of a number of biomarkers that allows us to classify based on biomarker data.

**Table 1:** **Differences between the standard and the network-based AD biomarker approaches**.

	AD biomarker	AD network-based biomarker (NBB)
Dimensionality	1-Dimensional, unsuited for multi-modal integration of heterogeneous data	N-Dissmensional, integrate multi-modal biomarkers in a common framework
Statistical classification	Classifier based on group differences between HC, MCI, AD	Supervised classifier for the assessment of risk disease in relation to large population data. Allows group risk classification based on individual-based risk measure built upon network biomarker parameters
Temporal scale	Temporal window of biomarker efficiency is not considered	Well suited for longitudinal studies by implementing computational models of network disruption effects in temporal windows, e.g., short/long term
Spatial scale	Study of selective vulnerability in region specific neuron classes, i.e., neuronopathy or network component specific, e.g., the precuneus in the DMN	Unbiased, NBB address large-scale distributed networks. Long rage disease spread shaped by network connectivity profiles, i.e., network-opathy (Comon, 1994)
Early diagnosis	Diagnosis of patients with overt dementia	Characterization of asymptomatic and prodromal stages. NBB can be used as surrogate end points and provide *in vivo* intermediate phenotypes of pathology
Preventive therapy	Inefficient for disease-modifying or preventive therapies, e.g., reduction of Aβ production has shown limited therapeutic impact	Potential for early diagnosis and disease-modifying therapies by detecting alterations in functional connectivity
Feature extraction	Absence of standardized quantitative metric for AD imaging biomarkers	Automated extraction of network parameters borrowing tools and methods from network theory

Our network-based approach in AD biomarkers differs from these approaches in that biomarkers are here characterized as structured objects, i.e., networks, in which the dependencies among the network components, i.e., individual biomarkers, need to be quantified via experimentation or computational simulation of the network dynamics. For a training set of diagnosed biomarker data, the computation of the generative biomarker network for each diagnostic class, e.g., *M*_H_, *M*_AD_ is a network structure discovery problem. The idea is to provide a structural model, i.e., a network of the training data set, i.e., biomarker data. For example, for a training data set of patients diagnosed into the categories healthy and AD, two networks – *M_H_, M_AD_* – are built. The nodes represent the random variables of the training set (biomarkers) and the edges represent the stochastic dependency between these variables. Dependency structures can be analyzed using Bayesian network models (Buntine, 1996). In the context of AD biomarkers, the network represents the dependency structure of the underlying distribution of any two biomarkers. For example, in Figure 1, the generative network *M*_H_, which contains a structural representation of the biomarkers dependencies in the subjects diagnosed as healthy, shows no dependency among biomarkers and only one biomarker, amyloid-beta deposition, reaches the threshold of positivity. In the *M*_AD_ network, the generative matrix of patients diagnosed as AD, we find stochastic dependency between all pairs of biomarkers except in fMRI and tactile.

The identification of the generative models *M*_H_ and *M*_AD_ from data is the result of statistical learning followed by model selection. It ought to be noted that when the amount of data – the number of diagnosed individuals – is small compared to the size of the model – the number of biomarkers – there are likely many candidate models that explain the data, and therefore the generative model provided by model selection may not be a good approximation of the underlying process. On the other hand, model selection is more likely to provide a good approximation when a large amount of data is available in models with a relatively small number of parameters. The number of candidate networks is super exponential of the number of model parameters, therefore small size models relative to the large data sample are preferable. For a discussion of the *p, n* (*p* = model size, *n* = data size) problem in statistics, see Gomez-Ramirez and Sanz (2013). The diagnosis of a new subject can be computed via the maximum probability of the biomarker configuration BM_s_ conditional to the generative models, *M*_H_ and *M*_AD_, max_G_ = (*M*_H_, *M*_AD_) P(BM_s_|G).

The utility of this approach will ultimately rely on its power to generate decision support systems to assist the physician in early diagnosis and symptomatic treatment. This work describes the blueprint for the construction of uncomplicated and cost-effective tools for the identification of disease’s signatures, based on a new understanding of biomarkers as multidimensional objects, i.e., networks. Thus, biomarkers can be seen here as the heterogeneous building blocks in network-based models.

Conceptually, the work flow for the implementation of decision models based on the theoretical framework described here can be divided into three phases: (1) data extraction for biomarker selection, (2) network-based model building, and (3) model validation using classification algorithms. The first phase is intrinsically hypothesis driven. Quantities susceptible to work as biomarkers are selected experimentally or via public repositories such as the ADNI initiative. In the second phase, the interdependencies among biomarkers are studied quantitatively. The idea is to understand how the different biomarkers act together within a network model that can be further characterized in terms of network parameters such as clustering or modularity. As a result, generative models of diagnostic categories, e.g., *M*_H_ and *M*_AD_ are built. In the last step, new subjects can be diagnosed via the maximum probability of the biomarker configuration for a new subject s (BM_s_) conditional to the generative models, max_G_ = (*M*_H_, *M*_AD_) P(BM_s_*|*G). Thus, in essence, this approach can be seen as a supervised classifier that allows us to assess the clinical value of the network models built upon heterogeneous and structured biomarker data. It ought to be remarked that the Bayes’ theorem allows us to calculate the posterior probability P(G|BM_s_) or the updating of probabilities from an experiment that results in the biomarker values BM_s_. Generally speaking, by increasing the sample size it is possible to reduce the importance of the prior distribution, P(G), which is particularly difficult to specify, and represents the uncertainty about the network structure before the data are examined (Migon and Gamerman, 1999).

## Conclusion

The network-based biomarker approach described here is in compliance with the new emerging paradigm of network medicine (Barabási et al., 2011). In this respect, network medicine, in order to be successful, must offer healthcare professionals not only a conceptual framework, but also comprehensive methodologies and a practical toolkit able to address the challenges and limitations in AD biomarkers research in new ways. New classification methods, such as support vector machine (SVM), have proven to be effective for the identification of MCIs from normal aging using resting-state functional connectivity data (Wee et al., 2012). Bayesian network analysis of effective connectivity show differences in the DMN between AD and healthy controls and could be used in the future as a biomarker (Wu et al., 2011).

The development of efficient tools for use in clinical diagnosis and monitoring of disease progress require the improved use of already known biomarkers and new methods of biomarkers discovery. There is a strong need for objective- and quantitative-based biomarkers of use in asymptomatic and prodromal stages of AD. The systemic understanding of the interactions between biomarkers can be seen as statistical learning followed by a model selection problem. The inclusion of functional imaging biomarkers in the clinical diagnoses of AD necessarily passes over the standardization of imaging protocols and quantitative metrics. In this respect, the network-based biomarkers approach presented here goes beyond the current emphasis on the study of the relationship between specific networks (e.g., DMN) and molecular biomarkers (e.g., amyloid-beta) to learn dependencies between biomarkers from heterogeneous data implemented as a graph, where the nodes are biomarkers and the edges represent the stochastic dependency among the biomarkers.

There are, however, challenges that are not addressed here. For example, the review has focused on the integration of predetermined biomarkers, but biomarker selection is a standing problem in AD research. Non-linear relationships between biomarker measurements and disease severity, and handling sparse observations constrain biomarker prediction. Alterations in functional connectivity may play a key role in detecting signatures in pre-symptomatic and prodromal stages. However, functional imaging related biomarkers have so far focused on alterations in intrinsic connectivity networks and the co-occurrence of protein deposition within those networks. Quantified and standardized metrics for AD neuroimaging biomarkers and a system-level understanding of the dependencies among the existing biomarkers are still missing. The network-based approach introduced here aims to bridge this gap by providing a statistical framework able to learn structural representations of biomarkers interactions from biomarker data of previously diagnosed patients. To fully capitalize on the large amount of data that big data science projects are bringing to AD research, a new mathematical framework for finding effective combinations of multi-modal biomarkers is sorely required. Biomarkers deployed as network models rather than as quantities will foster our understanding of disease, paving the way for a predictive, preventive, and personalized medicine.

## Conflict of Interest Statement

The authors declare that the research was conducted in the absence of any commercial or financial relationships that could be construed as a potential conflict of interest.
